# Health in Africa

**DOI:** 10.1038/s41467-024-45268-1

**Published:** 2024-02-01

**Keywords:** Public health, Health sciences

## Abstract

Africa is undergoing a demographic transition^1^ that has led to significant reductions in the number of individuals living in extreme poverty, and to positive shifts in related health outcomes, across its diverse populations^2^. Building on these successes requires a consideration of intersecting factors that impact health metrics, which is the focus of the United Nations Sustainable Development Goals^3^. To support researchers in their efforts towards reaching these goals, *Nature Communications, Communications Medicine* and *Scientific Reports* invite submissions of papers that advance our understanding of all aspects of health in Africa.

Since 2000, Africa has made historic progress in improving health metrics across all age groups^4^. The WHO estimates that the region successfully reduced the number of malaria-, HIV-1-, and diarrhoeal-attributed deaths by 66%, 57% and 52%, respectively, and achieved sharp declines in under-5 mortality rates. At the same time, the overall average healthy life expectancy in Africa rose by 3 years, the most significant increase of any global region, and the gap between African countries with the highest and lowest healthy life expectancies reduced from 27.5 to 22.0 years^5^. While Africa has the youngest population structure in the world, by 2050, 163 million people on the continent will reach the age of 60^6^, up from 43 million in 2010, and the population is expected to reach 2.8 billion^7^. These successes have significantly contributed to its economic growth since 2000^1,7^ as more individuals are now reaching working age, and more women have entered the workforce^8^.

The current state of health in Africa is complex. The region has some of the world’s highest rates of preventable neonatal^9^ and maternal^10^ mortality, and deaths from infectious diseases^11^, antimicrobial-resistant infections^12^, and malnutrition^13^. Its recent economic successes, however, have brought lifestyle changes including rapid unplanned urbanisation^14^, a rise in both alcohol consumption^15^ and tobacco usage^16^; and an epidemic of non-communicable diseases that now account for over 37% of all deaths on the continent^17^. As life expectancies among African populations rise, the number of individuals living with multiple chronic health conditions (multimorbidity)^18^ is increasing. This phenomenon is only just beginning to be understood but, recent studies have shown that in Africa, multimorbidity is significantly correlated with urbanisation, female sex, being older than 50 years of age, and higher educational attainment^19–21^. The effects of rising multimorbidity on Africa’s health systems are yet to be determined; however, they emphasise the need for public health programmes, which have traditionally been focused on single diseases, to move to being able to diagnose and manage patients with intersecting conditions^22^.

Africa’s current and future health security will depend on many factors, which include the need for increasing the capacity for both specialised healthcare and research to address the aforementioned challenges^23,24^, and the need for reducing reliance on importing life-saving diagnostics, treatments, and medical technologies. The latter, especially, is the biggest component of African healthcare spending^5^ but it comes with significant risks as revealed during the Covid-19 pandemic, when it was one of the last global regions to receive life-saving vaccines against SARS-CoV-2, and routine childhood immunisation rates in Eastern, Southern, West, and Central Africa fell to the lowest of all UNICEF regions^25^, ranging from 69% to 74%. Climate change is also significantly impacting Africa’s health security. Between January and October 2022, the Horn of Africa experienced its fifth consecutive drought and reported outbreaks of anthrax, measles, cholera, yellow fever, chikungunya, meningitis, and other infectious diseases, which accounted for more than 80% of all acute public health events. The drought led to heightened food insecurity and to 4.5 million new climate refugees searching for food and water, which made them more vulnerable to both disease outbreaks and malnutrition^26^.

African-led initiatives will be critical in addressing these challenges and shaping the region’s health future^27^. Organisations including CDC Africa^28^ and Clim-Health Africa^29^ are already leading the way by working to increase Africa’s capacity to manufacture multiple life-saving vaccines and building regional capacity for delivering training in climate impacts on health to local public health practitioners, respectively. Through such initiatives, Africa will build on its economic, green and health achievements to make the African Union’s Agenda 2063^30^ a reality.

With the launch of this collection, we invite submissions of original research and commentary that advance our understanding of all aspects of health in Africa as outlined in its scope. At *Nature Communications*, primary research articles of particularly high relevance to the UN Sustainable Development Goals^3^ may be highlighted through a new Policy Summary feature that will discuss the practical implications of the discoveries for policy makers.

Authors wishing to submit to this collection should select the option for the “Health in Africa” collection on our submission system. We will require all potential authors, reviewers, and our editors, to adhere to our editorial policies by complying with the Global Code of Conduct for Research in Resource-Poor Settings^31^. We are particularly interested in receiving original research and commentary from authors based in Africa and are committed to supporting such authors with respect to our article processing charges. We encourage all prospective authors to contact us if they have any questions regarding this.
